# Anterior Upper Teeth Golden Proportion Analysis with Millimetric Templates: An Invention Developed at Londrina State University

**DOI:** 10.1155/2022/1520812

**Published:** 2022-12-05

**Authors:** Adriana De Oliveira Silva, Hebert Samuel Carafa Fabre, Wagner José Silva Ursi, Márcio Grama Hoeppner, Anna Laura Morais Do Amaral

**Affiliations:** Londrina State University, Londrina, Brazil

## Abstract

In order to achieve aesthetic and harmonious smile results, the use of anterior upper teeth golden proportion concepts represents reliable and scientific based guidelines. However, measuring, recording and analysing teeth and smiles biometric values proves to be a clinical and laboratory routine chalenge, once it is time consuming and demands additional especific math calculus or formulas. The aim of this paper is present an invention, “anterior upper teeth golden proportion millimetric templates,” a set of instruments fabricated in order to achieve precise and fast millimetric measures, once they present predefined geometrical drawings and diagrams. Currently, when planning aesthetic tooth size modifications treatments, tools are used as pachymeters, dry edge compass, or some softwares. Most of times this strategy relies on professional expertise and intuiton, which shows to be limitating factors with the need of trial-and-error training and an accurate critical and artistic sense. Unfortunately, this is not inherent to all professionals, especially undergraduate dental school students. Thus, the templates bring differentials and advantages, being versatile and convenient, allowing countless clinical and laboratory uses, even over a cellphone or a computer screen. An excellent diagnostic aid providing ideal teeth proportions and positioning, increasing the chances of success in dental treatment planning.

## 1. Introduction

The golden proportion is widely found in nature, being defined as a math formula to achieve correspondent harmony between two uneven parts, resulting in a proportion of 1,618 : 1. This same proportion can be largely used when establishing aesthetically pleasant dental relations, achieving balance between face and teeth [1].

Therefore, the smile when in a front view, is considered to be more aesthetically pleasant, if each tooth is approximately 61.8% the size of the immediately before teeth. The central upper incisor width must keep a golden proportion to the lateral incisor width, which in the same way has to keep it to the canine [2], in order to meet the requirements of recurrent regressive symmetrical proportion, resulting in balanced and harmonic smiles [3].

The use of the golden proportion in dentistry results in dominance of the maxillary central incisors, favoring the aesthetic effects of large, clearer and brighter teeth, reducing the feeling of monotony in the smile, caused the closer the widths of the lateral incisors and canines are in relation to the central incisors [3].

Currently, technological evolution has brought several advances and innovations in the field of dentistry, which has made it possible to plan in a fully digital way. Thus, for orthodontics, orthognathics and plastic surgery, several extremely important methods of evaluation of the soft tissue profile have been developed, including direct anthropometry, standardized 2D profile photography, 3D profile photogrammetry, standard lateral cephalometry, 3D stereophotogrammetry, direct analysis of soft tissues by Cone Beam Computed Tomography (CBCT) [4] as well as the 3D Total Face Approach cephalometric analysis method that allows the identification of skeletal disharmonies, their extensions and classifications for later determination of the success or failure of the correction [5].

Likewise, for aesthetic rehabilitation dentistry there are computer software programs for digital smile design (DSD), used as objective aesthetic analysis and, consequently, virtual planning through photographs and/or scanned models of patients in order to obtain reliable results. These systems have the disadvantage of requiring training for their use, in addition to being limited in terms of facial aesthetic parameters, focusing on dentogingival and dental aesthetic parameters [6]. Furthermore, they have a high acquisition cost because they operate on subscription licensing platforms [7], so despite being innovative tools, many times, in clinical routine, they become unfeasible because they require additional time and greater technological knowledge. Still, even with the planning carried out digitally, the transfer of this information to the clinical situation is a chalenge and the use of tools and instruments in practice becomes fundamental.

The principles that govern dental proportionality are not absolute in natural dentition. The proportions may vary between populations and should not be generalized, precisely because of their subjectivity and mutability [1, 8]. With this, there is a need for devices that suit the dentist to frame, display and measure the dimensions and characteristics in a logical and rational way, quickly and without improvisation, justifying the creation of the instruments in question.

The golden proportion however is only one of many factors involved in the smile design, representing one diagnostic tool in evaluation and a clinical guideline to be followed in the aesthetic rehabilitation of anterior teeth [9].

The aim of this paper is to present the invention “anterior upper teeth golden proportion millimetric templates” [10] developed by the londrina state university dental course additional formation program.

## 2. Materials and Methods

**Figure 1:**
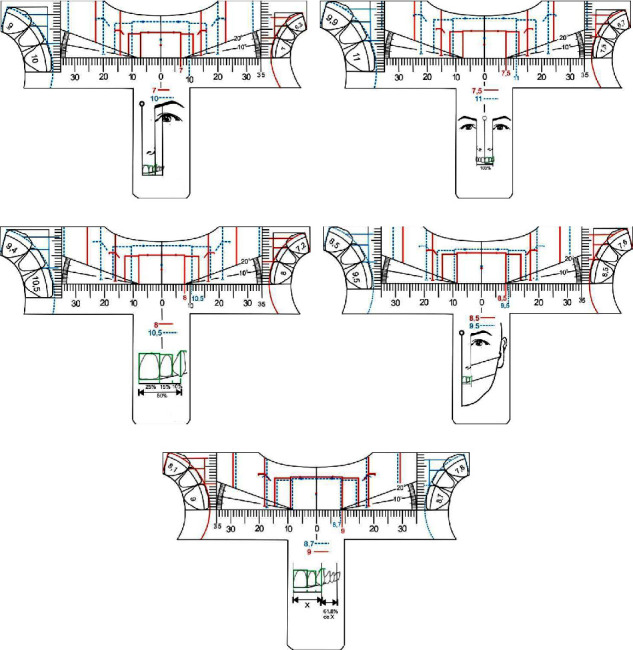
Frontal view illustration of the five golden proportion analysis millimetric templates.

The teeth golden proportion analysis templates [10] were developed after a intense bibliografic survey on tooth proportion and are caracterized by a set of 5 different translucent “*T*” format templates (Figure 1). Each item presents the description of values for two dominance sizes of the upper central incisor. Thus, ruler *A* comprises the golden ratio values for maxillary central incisors with widths of 7 mm, represented by continuous lines and maxillary central incisors with widths of 10 mm, represented by dotted lines. Likewise, ruler *B* comprises values of maxillary central incisors of 7.5 mm and 11 mm, *C* maxillary central incisors of 8 mm and 10.5 mm, *D* maxillary central incisors of 8.5 mm and 9.5 mm and, finally, *E* with 8.7 mm and 9 mm maxillary central incisors, arranged, so that, there is no overlap of lines and compromised visualization. The values were supported by biometric research and specific mathematical calculations, in order to cover 10 sizes of central incisors, in addition to the possibility of intermediate values obtained through millimeter scales [10].

**Figure 2:**
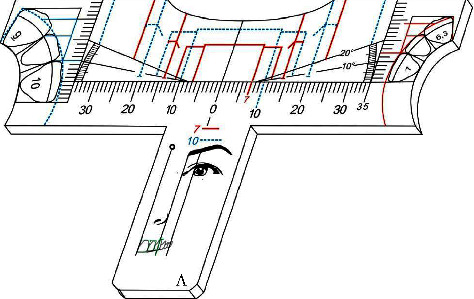
Perspective view illustration.

They must be fabricated out of crystal translucent acrylic, glass or plastic, or even another rigid or semi rigid material, smoothly finished, friendly decontamination and reusable if necessary. Its thickness may vary according to the fabrication material, being represented in perspective (Figure 2).

**Figure 3:**
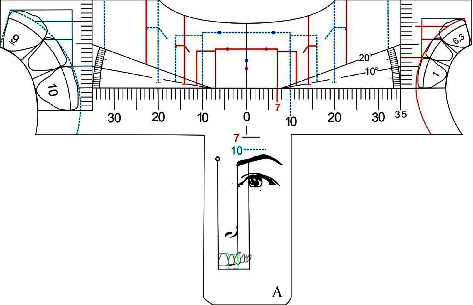
Front view illustration of one template showing metric scales and angles, diagrams and schematic drawings.

All rulers or templates present a horizontal millimetric scale, and in both end two vertical millimetric scales. Also, a protractor to measure angles up to 20 degrees to make it possible to check the inclination of the functional aesthetic occlusal plane, around 10°, parallel to the Camper plane, a line that passes from the tragus to the ala of the nose. They also bring different drawings and lines in order to facilitated the use, showing the respective golden proportion width for central incisors, lateral incisors, canines and buccal corridor bilaterally.  Figure 3 illustrates ruler *A* showing the metric scales, protractors, diagrams and drawings mentioned above (Figure 3).

**Figure 4:**
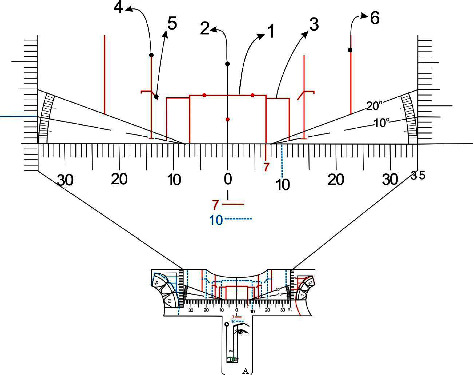
Anterior upper teeth golden proportion diagram illustration. (1) Upper central incisor framing rectangle; (2) facial midline; (3) upper lateral incisors rectangle; (4) vertical line for the canines; (5) canine proportion gingival line; (6) frontal general view of buccal corridor apparent width and posterior teeth lines.

On Figure 4 we defined the golden proportion diagram presented in all five templates. It is composed by two rectangles for both upper incisors that show an individual proportion of 80% in width for a 100% in height. Each incisor dominance measurement will be represented by continuous or dotted lines drawings and different colors in order to facilitate visualization (Figure 4, 1). At the center of the ruler we will find the midline reference (Figure 4, 2) and from the distal of the central incisor rectangle the protractor line initiates in order to check and verify the oclusal plane inclination comprede to the Camper plane.

**Figure 5:**
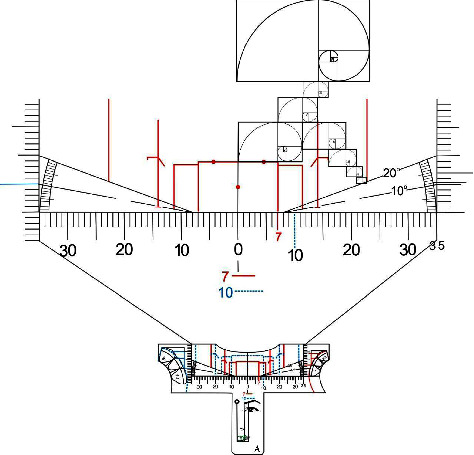
Anterosuperior presentation of the golden proportion concepts checked by the conventional golden proportion graphic scheme lines.

The rectangles that represent the upper lateral incisors are 61.8% in width of the upper central incisor rectangle and approximately 0.5 mm shorter in height to achieve an aesthetic concave cervical profile [3] (Figure 4, 3). For the canines rectangles were not drawn due to their positioning in the arch curvature compared to the central and lateral incisors, and also to guide the analysis by considering the exposure of the gingiva on the mesial, aiming to reduce the sensation of narrowing that would occur if they were represented by closed rectangles, thus, vertical lines drawn to the upper edge of the ruler keep the proportion of 61.8% with the lateral incisor (Figure 4, 4), in addition to slanted lines on the gingival following the geometry and position of the gingiva (Figure 4, 5). The canines, when they are not worn, keep the equivalent height of the clinical crown with the maxillary central incisors, but they are positioned superiorly, so that the incisal line follows the line of the lower lip when smiling, ideally having a “deep plate” design [3].

Due to variations, its apex may be positioned inferiorly, so on the ruler there is a region of discontinuity in the protractor line, allowing the canine to exceed it, without, however, reaching the same plane as the central incisor.

**Figure 6:**
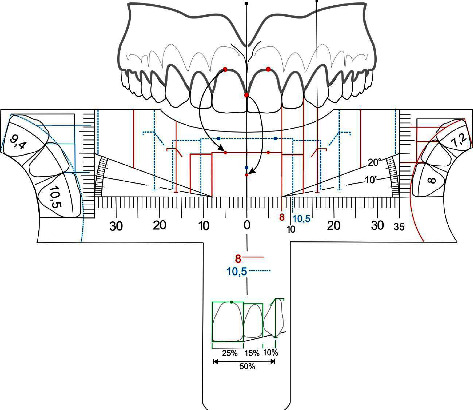
Upper dental arch illustration showing teeth position and inclination, the gingival zeniths and the upper central incisor papilla positioning.

Also, there are lines of apparent width of the posterior gradation index, golden proportion perceptible in the frontal analysis of the distal of the canine, first and second maxillary premolars and mesial part of the maxillary first molar, so that the visibility of these teeth gradually decreases, in a regressive proportion [3]. Thus, the space from the marking of the lines, bilaterally, to the labial commissure during the smile corresponds to the buccal corridor (Figure 4, 6).

At Figure 5, it can be contemplated the perspective of the distal canine limit in order to check the regressive posterior teeth appearance, as well as the apparent buccal corridor width lines, still regarding the golden proportion concepts.

The position and inclination of teeth in the dental arches (Figure 6) are responsible to determinate the gingival zeniths, represented by a small circle for the upper central incisors. However, for the maxillary lateral incisors, due to their broad anatomic variation, the zenith point is not presented. The upper canines zeniths are, most of times, higher than the lateral incisors zeniths, and approximately at the same height as the central ones, making the cervical line convex in relation to the occlusal plane. These are not presented in the invention, however, the cervical contour curvature normally matches the line drawn for the canines.

When analysing the dental papilla, it is possible to indicate an ideal height, once they fill up half of the size of the central incisors in normal conditions. This pattern is expected to be repeated for upper lateral incisors and canines. However, it was chosen to represent its position only between the central incisors.

**Figure 7:**
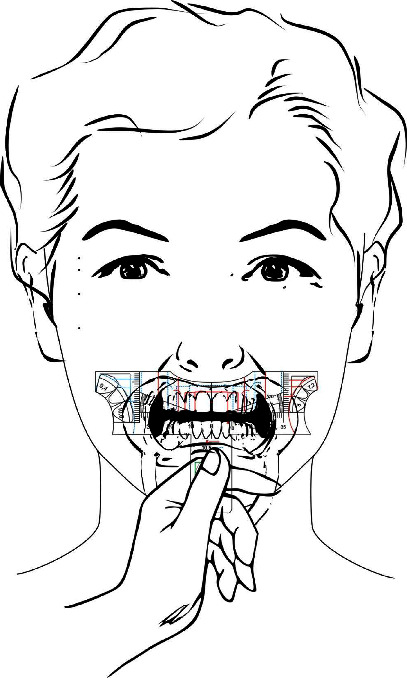
Frontal view illustration of the invention handling golden proportion analysis.

**Figure 8:**
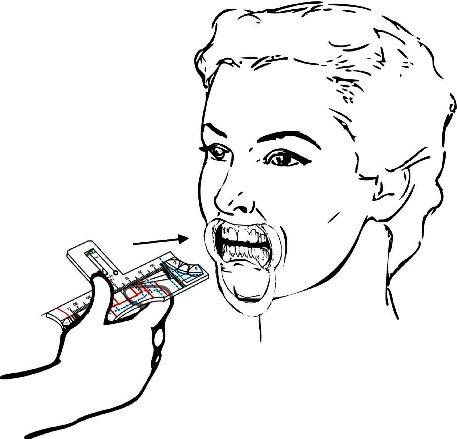
Invention handling illustration of the ideal individual proportion drawings on the lateral aspect of the ruler.

The models can also be used vertically and frontally to the upper incisors (Figure 7), and horizontally, once it is presented in the lateral portions of the template an oclusal diagram of upper anterior teeth in golden proportion, allowing the understanding the individual width of each tooth, what makes the invention a complete dental reference tool (Figure 8).

In order to draw individual proportions or real aesthetic proportions the relation height versus width of each tooth was used. For the central incisor the width represented 80% of the height, for the lateral incisor 69% and for the canines 72%. We also considered that the central incisor golden width is equal to its real individual width, once its alignment is parallel to the coronal plane. In the other hand, the lateral incisor golden width represents 80% of its real individual measure and the canines 44.5% (Table 1).

**Figure 9:**
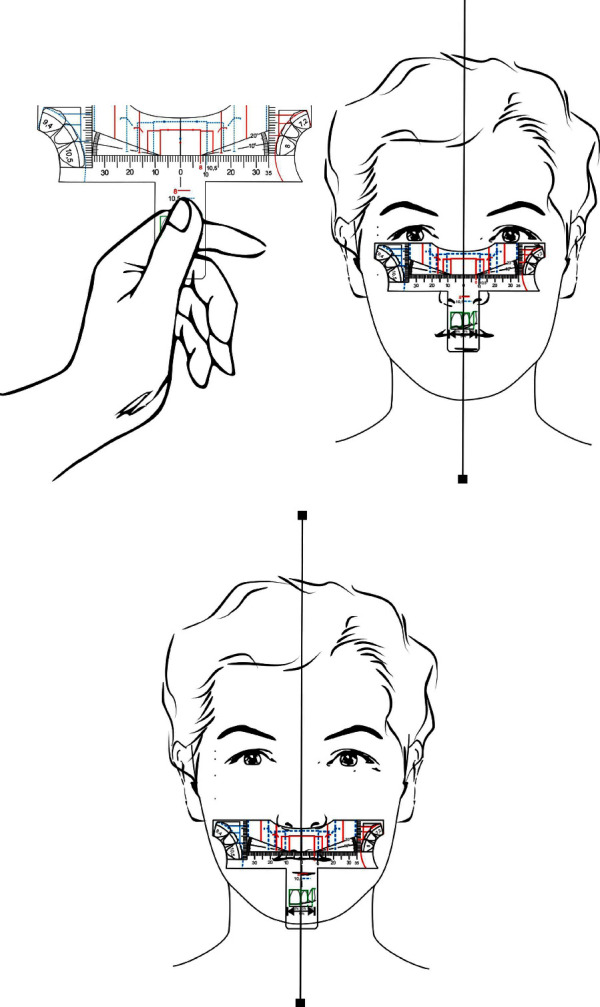
In (a) manual handling of the invention; and in (b) and (c)–illustration of the invention uses in order to choose the ideal option using as parameters intercantal and interalar distances, being the patient's face serious and at rest.

**Figure 10:**
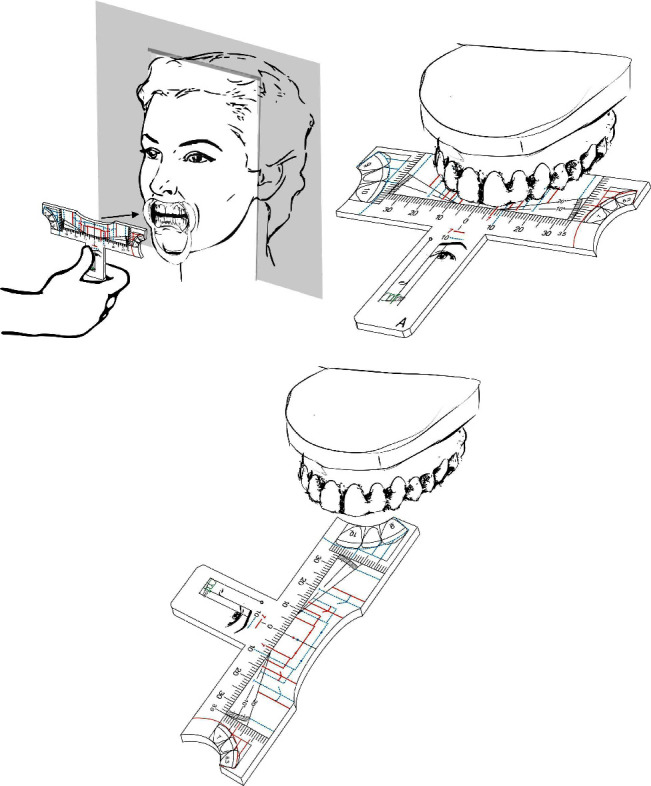
In (a)–tooth analysis invention handling illustration, being the template parallel to the coronal plane, and ideally using oral retractors for better visualization. In (b)-the invention handling illustration over a desk when using plaster dental casts or in (c)–using the ideal individual proportion drawings on the lateral of the template.

The invention can be used manually, evaluating the ideal template for each case comparing the intercanines distance markings to the intercanthal and interalar distances (Figure 9). In vivo teeth analysis can be performed with the aid of a mouth retractor and the template must stand parallel to the frontal (coronal) plane and aligned to facial midline. In cases where there is no coincidence between the dental midline and the facial midline, there is the option of carrying out the restorative treatment following the midline of the face, when this discrepancy is not so significant and allows for a favorable aesthetic result or not following it, in cases of greater complexity and disharmonies, aligning the teeth within the intercanine distance. Thus, the analysis using the rulers allows the dentist to become aware of existing imbalances. When studying plaster dental models it is advised to perform the analysis over a work bench or using the drawings and individual proportions on the lateral of the ruler (Figure 10).

## 3. Results

The devices here presented offer easy way to measure dimensions, angles and tooth proportions following the golden concept in a logic and rational way. Once the invention presents a set of clear and plane rules, it allows the professional to visualize the framework, obtaining more detail perception sharpness and alterations identification, on a precise manner, avoiding improvisations, and can be applied by the dental and medical industry.

## 4. Discussion

Currently, in order to approach smile aesthetics and harmony the most used solutions are professional experience, intuition and consulting patient desires, once dental aesthetic profile satisfaction is mandatory in order to achieve more satisfactory results [11].

These solutions face limiting factors as training necessity based on right or wrong approach and accurate critical and artistc sense which, unfortunately, is not inherent to all professionals. Besides this, there is a broad variation on personal teeth appreciation among patients that must be taken in consideration [11].

Once the invention presents a millimetric scale facilitates the consultation of measures which, eventually, are not contemplated by the golden proportion rectangles geometric drawings, overcoming the limitations of the instruments until then available in the dental market, which is extremely important since the size, morphology of teeth and arches and their individual characteristics differ from one population to another and, consequently, the aesthetically pleasing parameters change [12]. The representation of height/width diagrams allows the verification of differences related to sexual dimorphism that would not be possible with the use of tooth-shaped templates. In addition, the diagrams represent average dental values of clinical crowns that, as reported in the literature, present statistically insignificant differences for men and women [13].

Besides that, other advantages are the representation of height/width golden proportion and the contemplation of each individual tooth width. Its transparency allows inventions and attends both right and left sides, differentiating the invention from the other available tools and methods, such as the 1978 Levin's golden proportion scales [2], the stainless steel three-point compass that starting from the measurement of a given tooth with the first two tips, the third is automatically positioned during movement, respecting the “golden rule” [14], Mondelli's bilateral scales, consisting of nine colored acrylic grids [15], pachymeter, dry point compass or even school rules.

Although technological evolution allows softwares development that are capable of analysis digital photographs in computers and cell phones apps and, consequently perform digital dental planning, most of them present limiting factors as high cost and computing skills, in addition to presenting high acquisition costs as they operate on subscription licensing platforms [7].

Studies have found the absence of the golden proportion in different populations [9, 11, 12, 16–18], as they did not consider the distal portion of the canine in the frontal analysis of the smile. In the invention, canine teeth are analyzed according to their position in the arch and anatomical condition, being divided into a mesial segment and a distal segment. Thus, in frontal view, the canine is included as a whole, but analysing its mesial portion, in anterior golden proportion, and the distal portion, in posterior gradation, so that there is no sensation of arch narrowing [19].

The proportions have shown on the rulers provide information for the width of the maxillary anterior teeth. Therefore, the diagrams present can be defined by the golden symmetric recurrent proportion, essentially understood, but not limited, since changes in the apparent width calculation do not interfere with the height of the crowns, functional aesthetic occlusal plane, papilla position, zenith and cervical curve. It is foreseen, in the patent process, the possibility of variation in the chosen proportions, according to the population served, however, the golden proportion is the one that results in smiles closer to the preference parameters.

It is important to emphasize that in current highly competitive society, a pleasant appearance is capable of differentiate personal and professional success and failure [3]. However, it is worth mentioning that aesthetics is not absolute, by contrast, it is extremely variable and subjective, impossible to be numerically quantified, but evaluated jointly and harmoniously with the facial components [20].

## 5. Limitations

The golden proportion is not absolute and although there is disagreement about the ideal value for the calculations, there seems to be a consensus on the importance of a recurrent symmetric regressive proportion in the analysis of anterior maxillary teeth, whatever it may be, becoming fundamental for the aesthetics and harmony of the smile [3]. Therefore, despite the limitations, the golden ratio values and favors a pleasant dominance of the central incisors and when added, in the dental analysis, the value of the distal of the canine, also in golden proportion, generates balanced results and, consequently, of greater satisfaction.

## 6. Final Considerations

The invention, “anterior upper teeth golden proportion analysis with millimetric templates,” is a practical and versatile tool, which allow various forms of use, including facial harmonization studies, calibrated photographic records leading to accurate biometric studies, representing an excellent auxiliary diagnostic tool once posibilitate ideal teeth positioning and proportion, increasing the chances of successful planning. A partnership between the londrina state university and the indusbello company, signed in December 21 2021, will enable the fabrication and selling to the innovation products dental market, which will help dentists during dental aesthetic procedures.

## 7. Clinical Significance

The devices here presented offer easy way to measure dimensions, angles and tooth proportions following the golden concept in a logic and rational way. Once the invention presents a set of clear and plane rules, it allows the professional to visualize the framework, obtaining more detail perception sharpness and alterations identification, on a precise manner, avoiding improvisations, and also can be applied by the dental and medical industry.

## Figures and Tables

**Table 1:** Golden dental metric references based on upper central incisor width and height, in millimeters.

Teeth measured millimeters											
Upper central incisor		Upper lateral incisor			Upper canine					Posterior gradation index	Intercantal distance
Width	Height	Golden width	Height	Individual width	Golden width (mesial)	Golden width (distal)	Total apparent width	Height	Individual width		
7.0	8.75	4.33	7.61	5.25	2.67	1.65	4.33	8.75	6.30	8.65	28
7.5	9.38	4.64	8.15	5.63	2.86	1.77	4.63	9.38	6.75	9.27	30
8.0	10.00	4.94	8.70	6.00	3.06	1.89	4.94	10.00	7.20	9.89	32
8.5	10.63	5.25	9.24	6.38	3.25	2.01	5.25	10.63	7.65	10.51	34
8.7	10.88	5.38	9.46	6.53	3.32	2.05	5.38	10.88	7.83	10.75	35
9.0	11.25	5.56	9.78	6.75	3.44	2.12	5.56	11.25	8.10	11.12	36
9.5	11.88	5.87	10.33	7.13	3.63	2.24	5.87	11.88	8.55	11.74	38
10.0	12.50	6.18	10.87	7.50	3.82	2.36	6.18	12.50	9.00	12.36	40
10.5	13.13	6.49	11.41	7.88	4.01	2.48	6.49	13.13	9.45	12.98	42
11.0	13.75	6.80	11.96	8.25	4.20	2.60	6.80	13.75	9.90	13.60	44

## Data Availability

The references data used to support the findings of this study are included within the article. Once it is an invention, there is few data published about it, but the detais data used to support the findings of this study are available from the corresponding author upon request.
